# Impact of an In-House Pediatric Surgery Unit and Human Milk Centered Enteral Nutrition on Necrotizing Enterocolitis

**DOI:** 10.1155/2018/5042707

**Published:** 2018-11-13

**Authors:** Sonja Zamrik, Federica Giachero, Michael Heldmann, Kai O. Hensel, Stefan Wirth, Andreas C. Jenke

**Affiliations:** ^1^Department of Gynecology and Obstetrics, University Hospital Essen, Germany; ^2^EKO Children's Hospital, Oberhausen, Witten/Herdecke University, Germany; ^3^HELIOS University Medical Center Wuppertal, Department of Pediatrics and Neonatology, Germany

## Abstract

The importance for mortality and morbidity of an in-house pediatric surgery unit for premature infants with necrotizing enterocolitis (NEC) remains undefined. Data on 389 consecutive very low birth weight infants with a birth weight <1250 g admitted between 2009 and 2014 was retrospectively analyzed in two almost identical neonatal intensive care units. Epidemiological data (n=172 and n=217, respectively) were comparable. Incidence of NEC stage II+ was significantly higher in center 1 (15.1 versus 5.5%, n=18 versus 6). This correlated with a significantly lower rate of exclusive human milk feeding compared to center 2 (24.2 versus 59.3%). Probiotic treatment did not differ. Importantly, in case of surgery the length of removed intestine (49.9 versus 19.5 cm) and the rate of severe short-bowel syndrome (38.9 versus 0 %) were significantly higher in center 1 (no in-house pediatric surgery). Furthermore, long-term morbidity assessment revealed more impaired motoric (-4.2 versus -2.2 months, p=0.21) and psychologic (-4.3 versus -1.6 months, p=0.09) development in center 1. Mortality was similar in both centers.* Conclusions*. Short- and possibly also long-term morbidity of NEC is clearly associated with the presence of an on-site pediatric surgery unit. Enteral nutrition with human milk seems to be a strong protective factor against NEC.

## 1. Introduction

Necrotizing enterocolitis (NEC) is still one of the most devastating acute disorders in premature infants [1–4]. Despite a high research priority over the last 50 years, its pathophysiology is still unclear. However, over the last decade evidence accumulated that an exaggerated, excessive, and unrestricted immature intestinal host immune response might be the primary factor leading to NEC. This is possibly triggered by environmental factors such as formula milk [5–8]. Since the search for reliable diagnostic NEC-related biomarkers has been unsuccessful so far [9–11], the diagnosis still relies almost exclusively on clinical parameters and thus on the clinical expertise of the handling neonatologist and pediatric surgeon. Currently, intestinal resection is still required in up to 80% of premature infants with NEC stage II or III who are referred for surgical evaluation [12]. Similarly, mortality and morbidity, such as short-bowel syndrome or sepsis, are still high in these children in addition to the associated impairments in quality of life. Besides these individual problems, NEC is a major long-term social and financial burden for society [13].

In the German medical system, one of the requirements of a tertiary neonatal care center is the availability of a pediatric surgeon consultant at any time [14]. An on-site department of pediatric surgery is, however, not required [14]. Whereas there have been several trials aiming to evaluate the best primary surgical approach (laparotomy versus peritoneal drainage) [15, 16], there has not yet been a study to evaluate the effect of the on-site availability of a pediatric surgeon consultant on the outcome of premature infants with NEC.

In this retrospective study, we provide the first data on the effect of an on-site pediatric surgery department on the outcome of premature infants with NEC. Therefore, we compared clinical data of all premature infants with a birth weight <1250 g admitted between the 1st of January 2009 and 31st of December 2014 at two almost identical German neonatal tertiary care centers. The main difference was that center 2 included an on-site pediatric surgery department whereas center 1 cooperated with a pediatric surgery service located in another hospital within 40 kilometers distance.

## 2. Methods

### 2.1. Hospital Characteristics

Both tertiary care centers are responsible for approximately 500,000 inhabitants. On average, both neonatal services feature 250 annual neonatal admissions born in-house, approximately 50 out-born neonates, and 50 to 65 very low birth weight infants with a birth weight <1500 g. The Children's Hospital Oberhausen (center 2) comprises an on-site pediatric surgery department, other than the Children's Hospital Wuppertal (center 1). Here, the demand of pediatric surgical expertise was covered between 2009 and 2014 by cooperation with a 40 km distant pediatric surgery service, and infants requiring surgery were transferred to this unit.

### 2.2. Epidemiological Data

Medical, nursing, and laboratory records of all newborns with a birth weight <1250 g admitted to both neonatal services between 1st of January 2009 and 31st of December 2014 were reviewed. Anonymized data including date of birth, sex, gestational age, birth weight, maternal underlying disease, intestinal problems/surgery, applied medications, administration of intravenous/central catheters or nasogastric tubes, and Bayley scales at the age of 24 months were collected and analyzed. Also standard operating procedures were assessed. Severe short-bowel syndrome was defined as the dependence on parental nutrition at the corrected age of 6 months.

### 2.3. Statistical Analysis

Data were compared using the Mann-Whitney U test according to normality assumptions on univariate analysis followed by Bonferroni correction for multiple testing. Categorical variables were compared using Fisher's exact test. Statistical analyses were performed with GraphPad Prism, version 5.0. The study was carried out in accordance with the declaration of Helsinki and approved by the Witten/Herdecke Ethics Committee. Written informed consent was obtained from legal guardians where appropriate.

## 3. Results

### 3.1. Epidemiological Data in Both Centers

During the 5-year period from 2009 to 2014, 172 infants with a birth weight below 1250 g were born in center 1 and 217 in center 2. Overall, the mean birth weight was significantly lower in center 2 due to a higher percentage of small for gestational age infants. Also there was a trend for higher nicotine consumption in center 2 (Table 1). Otherwise, no relevant significant differences were found between both cohorts including relevant outcome parameters such as intraventricular hemorrhage (IVH), retinopathy of prematurity (ROP), or severe bronchopulmonary dysplasia (BPD) (Table 1).

### 3.2. Prevalence of NEC Was Not Associated with Probiotic Treatment but with Human Milk Feeding

The prevalence of intestinal complications was three times higher in center 1 compared to center 2. This was associated with an increased prevalence of NEC (Table 2).

Interestingly, the application of probiotics did not differ between both centers. Both centers used Infloran Berna® and started probiotic therapy when oral feeding tolerance reached 80 to 100 ml/kg/day with a dose of 1/2 capsule for infants <1000 g and 1 capsule in >1000 g body weight. When Infloran Berna® was unavailable due to a commercial supply shortness, both hospitals changed to Lactobacillus rhamnosus (LGG®). Feeding protocols between both centers were very similar. Enteral feeding was initiated on day 2 of life as trophic feeding and administered by bolus. Subsequently, enteral feeds were increased by 10-20 ml/kg/day as tolerated. Importantly, feeding with human milk was much more common in center 2 where 75.9% of the infants received human milk compared to only 30.8% in center 1. Moreover, transition from parenteral to enteral nutrition was slightly faster in center 2 (Table 3).

### 3.3. In-House Pediatric Surgery Is Associated with Improved Intestinal Morbidity but Not with Mortality

We further analyzed the management and outcome of patients with intestinal problems. The overall incidence of surgery due to NEC differed significantly between the centers, 10.4% versus 2.7% (p=0.002). This difference was much lower and even not statistically significant in the group of exclusively formula milk fed infants (6.7 versus 3.8%, p=0.72). Interestingly, there were no differences in the rate of surgery procedures in suspected NEC between both centers (69.2% (18/26) versus 54.5% (6/11); p=0.46). However, the length of removed intestine was significantly higher in center 1 (Table 4).

**Figure 1:**
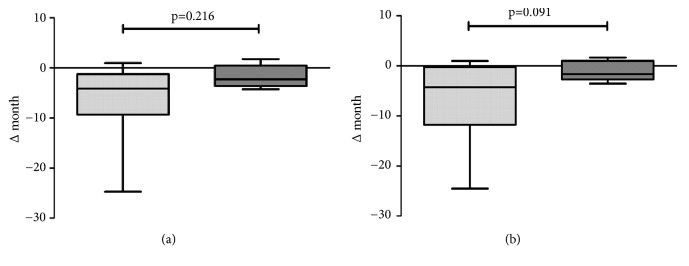
Bayley scales of premature infants with NEC. Delay in motoric (a) and behavioral (b) development in premature infants with NEC obtained at the corrected age of 24 months. Light grey (n=14) represents center 1 and dark grey (n=5) center 2.

As a consequence, among the 14 surviving NEC patients in center 1, 7 developed a short-bowel syndrome with 3 patients requiring parenteral feeding during the first 12 months of life. No cases of short-bowel syndrome occurred in center 2. Importantly, in contrast to the main cohort we did not observe any difference in patients who underwent surgery for NEC with respect to amount and quality of enteral feeds (Table 4). The only influencing factor seemed to be the time between being ordered to be NPO and first contact with the surgeon (Table 4). To assess long-term morbidity, we analyzed Bayley scales obtained at the corrected age of 2 years in all infants who had required surgery. Results of neurological testing were unavailable for 1 patient from center 2. Where Bayley scales could not be performed due to severe mental retardation, the developmental age was assessed by an experienced pediatric neurologist. No statistically significant differences between the centers could be detected regarding motoric and intellectual performance (Figure 1). However, we noted a trend to a better performance in center 2 with a less pronounced motoric (-2.2 versus -4.2 months, p=0.21) and psychologic developmental delay (-1.6 versus -4.3 months, p=0.09, Figure 1).

## 4. Discussion

With this study we provide the first data on the effect of an in-house pediatric surgery unit on the quality of clinical care for premature infants with NEC. Three main findings are of note: Firstly, as expected the threshold to contact the pediatric surgeon in the center with an in-house pediatric surgery unit was lower. Secondly, the in-house pediatric surgery department was associated with substantially improved short- and possibly also long-term morbidity. Thirdly, in the presence of identical probiotics supplementation human milk feeding was associated with a significantly reduced risk of NEC.

The current German guideline for tertiary neonatal care centers specifically includes the availability of a pediatric surgeon. Unfortunately, there is no clear definition of “availability” such as proximity and qualification [14]. Similar requirements and vague specifications can be found in guidelines from other countries such as the UK [17]. To date, there was no scientific evidence for the advantage of an on-site pediatric surgery department regarding the outcome of NICU patients. Our study clearly advocates for an early contact with a pediatric surgeon in case of abdominal symptoms in premature infants since time between NPO and first contact with a pediatric surgeon seems to be an essential factor influencing later mortality and morbidity (Table 4). Even though this cannot be causally explained owing to the retrospective nature of this investigation, the most reasonable explanation for this observation is a higher threshold of contacting the surgeon in the absence of an in-house pediatric surgery department. This idea is supported by the delayed time between onset of symptoms and involvement of the pediatric surgeon observed in our study compared to the center with an in-house pediatric surgery unit (Table 4). Importantly, it seems reasonable that this delay is a critical factor explaining the increased morbidity as reflected by a higher incidence of severe short-bowel syndrome and subsequent psychomotor developmental problems. Regarding short-term outcome this idea is supported by several recent studies demonstrating a direct correlation between depth of bacterial invasion and mortality in surgical NEC [18] and a highly protective effect of early laparotomy in terms of improved survival rate [19]. With respect to long-term morbidity it is well known that cerebral white matter injuries and neurodevelopmental outcome in premature infants are clearly correlated to frequency and intensity of postnatal exposure to inflammatory cytokines due to diseases such as NEC [20]. In conclusion, our data demonstrates an incremental value of an early contact with a pediatric surgeon for the outcome of VLBW neonates with NEC. However, even though a 24/7 immediate availability of a pediatric surgeon as it is only feasible with an in-house pediatric surgery unit is unquestionably the best option, we do not believe that this is a prerequisite. Instead we recommend an obligatory guideline defining the exact time point when the pediatric surgeon has to be involved. As a pragmatic approach this could be connected to the decision to place a premature infant on NPO. The time period between this decision and the first contact to a pediatric surgeon should be more clearly defined. Based on our data it is clear that more than 24h later is definitely too late, but the exact time period needs to defined in a larger cohort. From personal experience we believe that it should not exceed 8 hours.

Somewhat surprisingly, the incidence of NEC was very different between both centers, even though management guidelines regarding general handling, advancement of enteral nutrition, and probiotic therapy did not differ significantly. In fact, the application of probiotics followed the exact same standard operating procedure and both centers used the same probiotic agent obtained from the same manufacturer (Infloran Berna®). In the light of many studies demonstrating the preventive effect of probiotics [21] this finding was surprising. Interestingly, the only significant difference between both centers was the percentage of premature infants who received human milk (75.9 versus 30.8%). The vast majority of publications on NEC and probiotics did not include data on nutrition which at least in the animal model seems to play an important role in the pathogenesis of NEC [8]. Importantly, a recent study comparing NEC incidence in a single center before and after introduction of probiotics [22] failed to demonstrate an isolated probiotic effect. In this study, nutrition seemed to play a much more important role. Furthermore, a recent meta-analysis on the effect of probiotics in premature infants analyzed the studies considering the region where the studies have been performed. In this meta-analysis the authors could show a substantial preventive effect in all Asian studies (OR 0.31, 0.17-0.59). However, combined analysis of all European studies could not demonstrate a preventive effect (OR 0.88, 0.65-1.19) even though almost 2500 premature infants were included in this analysis [23]. Consequently, the prevention of NEC is not monocausal but much more complex and we need to consider environmental factors—notably the kind of enteral nutrition and human milk, respectively.

The main limitation is the retrospective nature of this study and the small sample size. This is a common challenge frequently reducing the significance in neonatal studies [24]. Furthermore, there are numerous influencing factors involved that may affect the incidence, treatment strategies, and ultimately the outcome for NEC patients. On the other hand, the two NICU centers included in this study are uniquely similar apart from the local pediatric surgery department, which particularly qualifies them to study this effect. Thus, a large-scale multicenter study may be prone to confounding bias and thus be equally conclusive. However, considering the reasonable advantage of an in-house pediatric surgery unit it is—from an ethical point of view—very questionable to withhold such treatment from premature infants. This is even more the case considering the data obtained in our study which makes a prospective study very difficult to design.

In summary, the results from this retrospective study comparing two neonatal tertiary centers with almost identical epidemiological and organizational parameters except for the pediatric surgery service clearly underscore the importance of an in-house pediatric surgery department for short- and long-term morbidity of premature infants with NEC. In addition, the data clearly advocates for the administration of human breast milk to premature infants.

## Figures and Tables

**Table 1:** Epidemiological and outcome characteristics.

	**Center 1**	**Center 2**	**p-value**
	**(n=172)**	**(n=217)**	
Gestational age (weeks as median, mean ± SD)	27 (27.34; 2.756)	27 (26.99; 2.75)	0.20
Birth weight (gram as median, mean ± SD)	955 (913.2; 243.6)	830 (856.6; 271.8)	0.03
Age of the mother (years as median, mean ± SD)	29 (29.56; 6.05)	32 (31.14; 5.91)	0.01
Nicotine consumption during pregnancy (%)	23.8	33.2	0.056
SGA (%)	27.3	38.7	0.02
Male gender (%)	52.3	47.4	0.35
Singletons (%)	76.8	65.9	0.02
Prenatal steroids	71.9	72.9	0.86
Amnion infection syndrome (%)	18.4	18.9	0.99
IVH (grade 0-2)	93.4	90.3	0.35
IVH (grade 3-4)	6.6	9.7	0.35
RPM (grade 0-2)	90.9	90.4	0.99
RPM (grade 3-4)	9.2	9.6	0.99
Severe BPD (grade 3)	5.1	4.0	0.79

**Table 2:** Prevalence of intestinal complications in neonates <1250 g.

	**Center 1**	**Center 2**	**p-value**
Incidence of intestinal complications (%)	17.4 (n=30)	6.6 (n=14)	**0.001**
FIP (%)	2.3 (n=4)	0.5 (n=1)	0.17
NEC stage 2 plus (%)	15.1 (n=26)	5.1 (n=11)	**0.0009**
Other intestinal complications (%)	0 (n=0)	0.9 (n=2)	0.51

**Table 3:** Enteral feeding regimens.

		**Center 1**	**Center 2**	**p-value**
Exclusively human milk (%)		24.2	59.3	**<0.0001**

Human milk combined with formula (%)		6.6	16.6	**0.0250**

Exclusively formula milk (%)		69.2	24.1	**<0.0001**

Amount of oral feeds in ml/kg/die (median, mean ± SD)	on day 5	49 (50.33; 29.97)	45 (47.65; 28.69)	0.4962
	on day 10	102 (101.1; 44.68)	99 (98.19; 42.01)	0.6072
	on day 15	139 (117.3; 59.58)	145 (135.5; 40.08)	**0.0032**

Prophylaxis with probiotics (%) on day 14 of life		76,5	83,3	0,99

**Table 4:** Mortality and morbidity of neonates who underwent surgery due to necrotizing enterocolitis.

		**Center 1**	**Center 2**	**p-value**
		**(n=18)**	**(n=6)**	
Gestational age (weeks) as median, mean ± SD		25 (24.9; 1.54)	27.5 (27.35; 3.98)	0.04

Birth weight (gram) as median mean ± SD		759 (746.4; 230.7)	775 (805; 362)	0.65

SGA (%)		22.2	50.0	0.31

Male gender (%)		44.4	66.7	0.64

Singletons (%)		83.3	100	0.55

Prenatal steroids (%)		77.8	50.0	0.31

Amnion infection synd. (%)		25.0	33.3	0.99

IVH (grade 3-4, %)		12.5	33.3	0.29

RPM (grade 3-4, %)		26.7	33.3	0.99

BPD (grade 2-3, %)		46.2	16.7	0.60

Death (%)		22.2	0	0.28

Time between NPO and first contact with surgeon (days) as mean ± SD		1.824 (2.4)	0.1667 (0.6)	**0.008**

Length of removed intestine (cm) as median, mean ± SD		39 (49.96; 36.7)	11 (19.5; 17.25)	**0.043**

Abdominal surgical procedures per patient as median, mean ± SD		3 (2.6; 0.8281)	2 (2.6; 0.8944)	0.99

Short bowel syndrome (%)		38.9	0	**0.03**

Exclusively human milk (%)		27.7	33.3	0.99

Human milk combined with formula (%)		27.7	33.3	0.99

Exclusively formula milk (%)		44.4	33.3	0.99

Amount of oral feeds in ml/kg/die (median, mean ± SD)	on day 5	46 (45.4; 20.3)	42.5 (41.7; 39.53)	0.74
	on day 10	78 (73.3; 45.7)	82.5 (82.5; 67.3)	0.73
	on day 15	71 (62.5; 71.6)	72 (84.5; 61.1)	0.33

Prophylaxis with probiotics (%) on day 14 of life		76,5	83,3	0.99

## Data Availability

The data used to support the findings of this study are available from the corresponding author upon request.

## Abbreviations

BPD: Bronchopulmonary dysplasia
FIP: Focal intestinal perforation
IVH: Intraventricular hemorrhage
NEC: Necrotizing enterocolitis
NPO: Nil per mouth
UK: United Kingdom
ROP: Retinopathy of prematurity
VLBW: Very low birth weight.

## Additional Points

*What Is Known about the Subject?* (i) Surgery is still a mainstay in therapy of necrotizing enterocolitis, but the exact importance for mortality and morbidity of a pediatric surgeon remains undefined. (ii) The currently most protective factors are probiotics and exclusively human milk diet, but their relative importance is still unclear.

*What This Study Adds*. (i) The availability of a pediatric surgeon is directly associated with long- and short-term morbidity in premature infants with necrotizing enterocolitis. (ii) The protective effect of exclusive human milk diet in preventing necrotizing enterocolitis seems to be more relevant than probiotic therapy.
